# Women Veterans’ perspectives, experiences, and preferences for firearm lethal means counseling discussions

**DOI:** 10.1371/journal.pone.0295042

**Published:** 2023-12-06

**Authors:** Evan R. Polzer, Ryan Holliday, Carly M. Rohs, Suzanne M. Thomas, Christin N. Miller, Joseph A. Simonetti, Lisa A. Brenner, Lindsey L. Monteith

**Affiliations:** 1 VA Rocky Mountain Mental Illness Research, Education and Clinical Center for Suicide Prevention, Aurora, Colorado, United States of America; 2 Department of Psychiatry, University of Colorado Anschutz Medical Campus, Aurora, Colorado, United States of America; 3 Firearm Injury Prevention Initiative, University of Colorado Anschutz Medical Campus, Aurora, CO, United States of America; 4 Department of Physical Medicine and Rehabilitation, University of Colorado Anschutz Medical Campus, Aurora, Colorado, United States of America; 5 Department of Neurology, University of Colorado Anschutz Medical Campus, Aurora, Colorado, United States of America; Uniformed Services University of the Health Sciences, UNITED STATES

## Abstract

**Aims:**

Firearms have become an increasingly common method of suicide among women Veterans, yet this population has rarely been a focus in firearm suicide prevention research. Limited knowledge is available regarding the preferences, experiences, or needs of women Veterans with respect to firearm lethal means counseling (LMC), an evidence-based suicide prevention strategy. Understanding is necessary to optimize delivery for this population.

**Method:**

Our sample included forty women Veterans with lifetime suicidal ideation or suicide attempt(s) and firearm access following military separation, all enrolled in the Veterans Health Administration. Participants were interviewed regarding their perspectives, experiences, and preferences for firearm LMC. Data were analyzed using a mixed inductive-deductive thematic analysis.

**Results:**

Women Veterans’ firearm and firearm LMC perspectives were shaped by their military service histories and identity, military sexual trauma, spouses/partners, children, rurality, and experiences with suicidal ideation and attempts. Half reported they had not engaged in firearm LMC previously. For those who had, positive aspects included a trusting, caring relationship, direct communication of rationale for questions, and discussion of exceptions to confidentiality. Negative aspects included conversations that felt impersonal, not sufficiently comprehensive, and Veterans’ fears regarding implications of disclosure, which impeded conversations. Women Veterans’ preferences for future firearm LMC encompassed providers communicating why such conversations are important, how they should be framed (e.g., around safety and genuine concern), what they should entail (e.g., discussing concerns regarding disclosure), whom should initiate (e.g., trusted caring provider) and where they should occur (e.g., safe spaces, women-specific groups comprised of peers).

**Discussion:**

This study is the first to examine women Veterans’ experiences with, and preferences for, firearm LMC. Detailed inquiry of the nuances of how, where, why, and by whom firearms are stored and used may help to facilitate firearm LMC with women Veterans.

## Introduction

In 2020, the sex- and age-adjusted suicide rate among United States (U.S.) Veterans was 57.3% higher than that of non-Veteran adults within the U.S. [1]. The magnitude of this difference was further pronounced among women Veterans, whose age-adjusted suicide rate (14.7 per 100,000) was more than twice that of women non-Veterans (6.8 per 100,000) in 2020 [2]. Understanding women Veterans’ use of specific suicide methods is critical for addressing this public health concern.

Among women Veterans, use of firearms as a method of suicide increased by 11.2% from 2001 to 2020 –an increase not observed to the same extent for Veteran men (4.8% increase) or non-Veteran women (2.1% decrease) [1]. Thus, in 2020, firearms were the most common method of suicide death among women Veterans and were used more among women Veterans (48.2% of suicide deaths) compared to women non-Veterans (33.3%) [1].

Firearm access and ownership are independent risk factors for suicide [3, 4]. As such, counseling at-risk individuals to reduce their access to potentially lethal means of suicide (i.e., lethal means counseling [LMC]) is an evidence-based suicide prevention strategy recommended by the National Action Alliance for Suicide Prevention and U.S. Surgeon General [5–7]. Given that firearms are, by far, the most common suicide method used among Veterans who die by suicide [1], the Department of Veterans Affairs (VA) has placed particular emphasis on firearm LMC [5, 8, 9]. The updated 2019 clinical practice guideline strongly recommended that clinicians assess for suicide risk factors, including “availability of firearms,” as part of comprehensive evaluation of suicide risk, in addition to “reducing access to lethal means to decrease suicide rates at the population level” [9]. LMC is also a key component of Safety Planning, a recommended individual-level intervention in which providers and patients collaboratively plan steps to take in a crisis to reduce suicide risk [10].

As such, understanding Veterans’ perspectives on firearm LMC is essential. Yet, *how*, *when*, *where*, and *why* Veteran patients may or may not find LMC to be acceptable is unclear. Systematic reviews of LMC utilization provide insights into LMC use and acceptability across various healthcare settings (see [11, 12]), yet little is known about how to optimize LMC among women Veterans specifically, nor have any firearm-related interventions been developed specifically for women Veterans. Rather, firearm-related suicide prevention efforts have espoused a gender-neutral approach [13] and have not taken into account gender-specific factors or the need for trauma-informed approaches that may be better suited for some within the women Veteran population [14]. Understanding women Veterans’ experiences with, perspectives on, and preferences for firearm LMC would align with the *Female Veteran Suicide Prevention Act*, passed by the U.S. Congress in 2016 and directing the VA to identify suicide prevention interventions that are both satisfactory and effective for women Veterans [15]. Additionally, hearing from women Veterans themselves about their perspectives, experiences, and needs regarding firearm LMC could help with tailoring LMC approaches for women Veterans to increase the acceptability and effectiveness of such approaches. Details regarding broader, contextual factors–including women Veterans’ perspectives with firearms, as well as their experiences engaging in firearm LMC–have not been readily addressed, yet such information could provide further insights into the curation and development of firearm LMC approaches for women Veterans.

Understanding women Veterans’ perspectives and experiences regarding firearm-specific LMC is also important considering gender differences in firearm access and ownership among Veterans. Although firearm-focused research with women has been limited, key differences have been identified. For example, women Veterans are less likely to own firearm(s) than Veteran men; however, women Veterans are more likely to reside in homes with firearm(s) that they do not personally own [16]. While Veteran men report a number of reasons for firearm ownership (and protection as a primary reason for ownership), women Veterans are more likely to report protection as their *sole* reason for owning firearms [16]. Additionally, in an initial qualitative study, women Veterans described interpersonal violence, such as sexual assault and/or sexual harassment during their military service, as an impetus for acquiring firearms and engaging in unsafe storage practices, in order to increase their sense of safety [17]; notably, this was not reported by Veteran men in a parallel qualitative study [18].

Importantly, women Veterans experience higher rates and increased severity of interpersonal violence compared to non-Veteran women and Veteran men [19, 20]. Further, women Veterans experience high rates of interpersonal violence (i.e., sexual or physical violence) across the lifespan, including during childhood, military service, and following military service [20–24]. Interpersonal violence experiences may prompt firearm acquisition or unsafe firearm storage and may pose unique challenges to using firearm LMC among women Veterans. For instance, if firearms function as a means of increasing personal safety among women Veterans, for whom interpersonal violence is highly prevalent, then women Veterans may be reluctant to engage in firearm LMC strategies, as reducing their access to firearms may cause them to experience heightened fear and distress of potential revictimization. However, for women Veterans experiencing intimate partner violence (IPV), their partners may use household firearms against them as instruments of control, to intimidate and threaten them; in such circumstances, the presence of household firearms further increases risk, both for suicide and homicide [25]. Determining how to approach firearm LMC in the context of interpersonal violence is essential to ensuring that firearm LMC is effective and responsive to the needs and experiences of women Veterans.

### Aims

To improve knowledge regarding how to take a gender-informed approach to firearm LMC with women Veterans, we examined women Veterans’ perspectives, experiences, and preferences regarding firearm LMC. We also sought to explore the extent to which women Veterans’ perspectives, experiences, and preferences varied based on history of interpersonal violence (i.e., sexual assault, IPV). These were examined among women Veterans who had experienced lifetime suicidal ideation or suicide attempt(s). These perspectives and experiences were expected to inform future research and delivery of firearm LMC among women Veterans.

## Method

### Participants

Inclusion criteria encompassed: age 18 to 89; self-identified gender identity or birth sex as female; prior active duty U.S. military service; eligible to receive Veterans Health Administration (VHA) care; used VHA care (at any point); personally owned firearm(s) or lived in a household with firearm(s) after separating from military service; lifetime suicidal ideation or suicide attempt(s), as assessed with items from the Self-Injurious Thoughts and Behaviors Interview [SITBI] [26]; and able to provide informed consent. Exclusion criteria included: currently serving on active duty, mobilized on Reserve or National Guard duty, or deemed to be at high acute suicide risk warranting hospitalization.

### Recruitment

Recruitment initiatives were multi-faceted. We mailed invitation letters to women Veterans enrolled in the VHA nationally, identified from the VA Corporate Data Warehouse (CDW; *n* = 3,655). We also sent mailings (*n* = 220) to women Veterans who previously participated in research with our center who consented to be contacted about future research. Additional recruitment methods included posting information about the study through electronic monitors in VHA facility waiting areas, at community events, informing healthcare providers about the study, and snowball recruitment (e.g., through other women Veteran participants). We used a purposeful sampling approach to ensure diversity in participants’ demographics (e.g., race, ethnicity, age, marital status), and geographic characteristics (e.g., region, rurality). Specifically, we monitored participant characteristics as study enrollment progressed, in order to identify gaps within the sample. Accordingly, later mailings emphasized specific Veteran characteristics (e.g., non-White, Hispanic; younger; married) to facilitate recruitment of a diverse sample. Lastly, to ensure that the sample included those whose firearm access was through personal ownership, as well as those who resided in homes with firearm(s) that other household members owned, ownership was queried during screening and considered when scheduling. Forty participants completed qualitative interviews (30 to 90 minutes in duration) between April 2021 and January 2022. Most participants were recruited through CDW (*n* = 22; 55.0%) or repository (*n* = 14; 35.0%) mailings, and a few through word-of-mouth (*n* = 4; 10.0%). Recruitment continued until we reached data saturation (when no new information was obtained from further interviews [27]).

### Procedures

All study procedures were approved by the Colorado Multiple Institutional Review Board (Protocol 19–2055) and the VA Eastern Colorado Healthcare System Research and Development Committee. Informed consent was obtained prior to initiating study procedures, via oral affirmation of consent by trained study staff. Appointments were conducted via phone or Microsoft Teams. After participants provided informed consent to participate, the University of Washington Risk Assessment Protocol (UWRAP [28]) was administered to ensure safety and appropriateness to participate. The qualitative interview (S1 Appendix) then commenced. Qualitative interviews were conducted by team members experienced in qualitative data collection (LLM, RM, SMT) and were audio-recorded. Recordings were transcribed by a professional transcription service and verified through cross-referencing completed transcripts against audio files.

Following the interview, an abbreviated version of the SITBI [26] was administered to assess for suicidal ideation and attempt and associated characteristics (e.g., timing, method). For descriptive purposes, we administered self-report measures to assess firearm behaviors and beliefs (based upon [16]); IPV (Extended-Hurt, Insult, Threaten, Scream [E-HITS] [29–31]), including past-year and lifetime; childhood physical and sexual abuse (using six items from the Adverse Childhood Experiences Study [32, 33]; adult sexual violence and physical assault (using two items from Sadler and colleagues [34]); military sexual trauma (MST; standard VA MST Screening Questions [35]; possible depression (PHQ-2 [36]); possible posttraumatic stress disorder (PTSD) (Primary Care PTSD Screen for DSM-5 [37]); alcohol misuse (CAGE-AID [38]); and sociodemographic and military service (PhenX Toolkit for Common Data Elements items [e.g., National Health and Nutrition Examination Survey, 2013–2014, Demographics Module; Behavioral Risk Factor Surveillance System 2018 Demographics; Military Service Demographics Protocol]). The appointment ended with the UWRAP post-assessment, followed by debriefing. Compensation of $50 was provided.

### Analytic plan

A bracketing exercise was first conducted among all study staff who participated in coding [39, 40]. Following a familiarization period that entailed reviewing transcripts, a pilot codebook was developed and beta-tested with a small sample of transcripts. Coders convened to deliberately code applications, definitions, and usage; identify potential issues with the pilot codebook; and modify it prior to continued use. A code hierarchy was developed that encompassed interview topics such as military service, experiences and utilization of VHA care, histories and perceptions of suicide among women Veterans, firearm use and access (including during heightened suicide risk), and experiences and beliefs regarding firearm LMC. Coders independently analyzed transcripts utilizing this codebook, then met as a team to discuss initial findings, discrepancies between coders, and modifications. This iterative process was utilized two additional times to achieve a high degree of consensus and mitigate biases. Two co-authors (ERP, CMR) employed a mixed inductive-deductive approach [41], allowing for relevant qualitative phenomena to emerge organically, alongside findings developed from prior theory (such as changes in firearm beliefs related to the Transtheoretical Model [42]). Consensus meetings occurred weekly, with team members iteratively deliberating and resolving differences in code applications and interpretations until there was agreement. One hundred percent of data were coded by at least one coder, and 60% were coded by two coders. We followed COREQ standards for reporting of qualitative research findings [43]. Qualitative analyses were performed using ATLAS.ti (v22.0.6). Quantitative analyses were performed using SAS, v9.4.

### Sample characteristics

To characterize the sample, descriptives are provided. The sample tended to be middle-aged (M = 51.33; SD = 12.38; range = 24 to 73). Additional sample descriptives regarding demographics, military service, and VHA use are provided in Table 1. All participants were cisgender women. The sample was diverse with respect to race and sexual orientation, although the majority of participants identified as White and as heterosexual. Marital status varied, with similar proportions of participants who were married/partnered or separated/divorced. The majority of participants did not currently live with a partner, very few lived with a minor, and only a minority reported current parenting responsibilities. Education and employment status varied, with the largest proportions of participants employed or disabled. Slightly less than half of participants had experienced lifetime homelessness. Participants were geographically diverse, including with respect to rurality/urbanicity. Most participants were from the South or West. Army was the most common branch of military service. Service era and deployment histories varied broadly. Most did not report prior combat tour(s). The majority had used VHA services in the prior year and were currently receiving VHA primary care. Many had obtained mental health care (primarily outpatient) in their lifetime, including in the past year and from a VHA facility.

**Table 1:** Demographic, military service characteristics, and VHA healthcare (N = 40).

Characteristic	*n*	%
**Gender**		
Woman	40	100.0
**Race** ^**a**^		
White	26	65.0
Black	9	22.5
Asian	1	2.5
American Indian/Alaska Native	5	12.5
**Ethnicity**		
Non-Hispanic	36	90.0
Hispanic	4	10.0
**Sexual orientation**		
Heterosexual	29	72.5
Gay	5	12.5
Bisexual	5	12.5
None	1	2.5
**Marital status**		
Married/couple	17	42.5
Separated/divorced	17	42.5
Widowed	2	5.0
Never married	4	10.0
**Lives with partner**	17	42.5
**Lives with minor**	7	17.5
**Parenting responsibilities**	6	15.0
**Education**		
High school or less	3	7.5
Some college	9	22.5
Associate’s degree	7	17.5
Bachelor’s degree	9	22.5
Master’s degree or higher	12	30.0
**Employment** ^**a**^		
Working now	15	37.5
Temporarily laid off/maternity leave/seeking	3	7.5
Retired	5	12.5
Disabled	17	42.5
Student	3	7.5
**Homelessness** (ever)^b^	17	42.5
**Rurality**		
Rural	8	20.0
Small Town	11	27.5
Med-Sized Town	4	10.0
Suburb	3	7.5
City	14	35.0
**Region**		
Northeast	2	5.0
Midwest	4	10.0
South	17	42.5
West	17	42.5
**Military service**		
Active duty	28	70.0
Active duty and National Guard	12	30.0
**Military branches** ^a^		
Army	21	52.5
Navy	6	15.0
Air Force	9	22.5
Marine Corps	6	15.0
**Service Era** ^**a**^		
OEF/OIF/OND	16	40.0
Desert Storm/Desert Shield	19	47.5
Post-Vietnam/Peacetime	20	50.0
Vietnam	3	7.5
**Deployments** ^**c**^		
None	21	52.5
Single	4	10.0
Multiple	15	37.5
**Combat tour(s) [any]**	15	37.5
**Use of VHA services in past year**	34	85.0
**Currently receiving VHA primary care** ^**d**^	35	87.5
**Lifetime MH care** ^**e**^	38	95.0

*Note*. Responses not endorsed by any participants (e.g., Native Hawaiian/Pacific Islander race, military service prior to 1964) are not included. MH = mental health; OEF/OIF/OND = Operation Enduring Freedom/Operation Iraqi Freedom/Operation New Dawn; VHA = Veterans Health Administration.

^a^ Totals could exceed 100% as response options were not mutually exclusive.

^b^ Reported previously (*n* = 16; 40.0%) or currently (*n* = 1; 2.5%) experiencing homelessness.

^c^ Deployed for the Gulf War (*n* = 7; 17.5%) and OEF/OIF/OND (*n* = 12; 30.0%)

^d^ Does not reflect primary care services provided outside VHA, but paid for by VA

^e^ Reported lifetime outpatient (*n* = 34; 85.0%) and inpatient (*n* = 14; 35.0%) MH care. Of those who reported lifetime MH care, the majority reported obtaining MH care in the past year (*n* = 27; 71.1%), most reported obtaining lifetime MH care at a VA facility (*n* = 35; 92.1%), and fewer reported obtaining lifetime MH care at a non-VHA facility (*n* = 21; 55.3%).

Most participants had experienced lifetime trauma and sexual violence (Table 2), including lifetime sexual violence, MST, childhood physical abuse, adult physical assault, and screened positive for lifetime IPV. Few, however, screened positive for IPV in the past year. The majority screened positive for current PTSD and current depression. Alcohol misuse was relatively infrequent. All participants had a lifetime history of suicidal ideation (not displayed); half reported a lifetime suicide attempt. Nearly two-thirds reported that they had considered firearms as a suicide method when thinking about suicide.

**Table 2:** Trauma, interpersonal violence, mental health, and suicidal self-directed violence (N = 40).

Characteristic	n	%
Lifetime trauma exposure	34	85.0
Lifetime sexual violence^a^	38	95.0
Military sexual trauma ^c^	34	85.0
Childhood physical abuse ^b^	30	76.9
Adult physical assault ^b^	24	61.5
IPV (lifetime) ^b^^,^^d^	33	84.6
Positive PTSD screen	34	85.0
Positive depression screen ^b^	26	66.7
Positive alcohol misuse screen ^b^	15	38.5
Lifetime suicide attempt(s) ^e^	20	50.0
Considered firearms during SI	25	62.5

*Note*. Responses not endorsed by any participants are not included in the table. Some participants who reported experiencing lifetime sexual violence (e.g., childhood sexual abuse, adult sexual assault, military sexual assault) did not report experiencing lifetime trauma exposure when completing the PC-PTSD-5. IPV = intimate partner violence; PTSD = posttraumatic stress disorder; SI = suicidal ideation; SA = suicide attempt.

^a^ Within the full sample, 27 (67.5%) reported childhood sexual abuse, 29 (72.5%) reported adult sexual assault, and 24 (60.0%) reported experiencing military sexual assault.

^b^ *n* = 39, due to one participant missing data

^c^ Within the full sample, 34 experienced military sexual harassment (85.0%) and 24 reported experiencing military sexual assault (60.0%).

^d^ *n* = 4 (10.3%) of the overall sample had experienced IPV in the past 12 months.

^e^ *n* = 15 (37.5%) of the overall sample had made multiple lifetime suicide attempts.

Sixty percent of the sample reported current firearm access, whereas the remaining 40% reported prior firearm access (Table 3). When examining personal firearm ownership 42.5% reported currently owning firearms(s) and 20.0% reported previously owning firearm(s). Nearly half of the sample reported that another household member currently or previously owned firearm(s), with 42.5% reporting that another household member currently owned a firearm and 5% reporting that another household member previously owned a firearm. Only half of participants reported that they had ever discussed household firearms with a healthcare provider; the majority of such discussion(s) had occurred with a mental health provider. Among the subset who currently owned firearm(s), most owned multiple firearms, all possessed handgun(s), and over half owned long guns. Protection from strangers, the most common reason for ownership, was nearly ubiquitous as a reason for ownership. The vast majority of firearm-owning participants indicated that owning a firearm made them feel safer. Firearm storage varied widely in terms of whether stored unloaded and locked. Nearly half of firearm-owning participants had handled a firearm in the past 30 days.

**Table 3:** Participant firearm characteristics (N = 40).

Characteristic	*n *	%
**Firearm access** ^a^		
Current (personal or household)	24	60.0
Past (personal or household)	16	40.0
**Personal firearm ownership**		
No, never	15	37.5
Yes, currently	17	42.5
Yes, previously	8	20.0
**Anyone else in household own a firearm**		
Yes, currently	17	42.5
Yes, previously	2	5.0
No	21	52.5
**Ever talked with a healthcare provider regarding household firearms and storage** ^b^		
Yes	20	50.0
No	20	50.0
**Subsample with Current Firearm Ownership (*n* = 17)**	*n *	%
**Current firearms owned** (*n* = 15)		
Single	5	33.3
Multiple	10	66.7
**Types of firearms owned** ^c^ (*n* = 17)		
Handguns	17	100.0
Long guns	9	52.9
**Reasons for owning firearms** ^c^ (*n* = 17)		
Protection from strangers	16	94.1
Protection from animals	9	52.9
Hunting/sporting use	8	47.1
Collection	4	23.5
Protection from people I know	3	17.6
Job/other	2	11.8
**Perceived safety due to owning firearms** (*n* = 16)		
Safer ^d^	14	87.5
Neutral	2	12.5
Less safe	0	0.0
**Storage of firearms** ^c^ (*n* = 17)		
Unloaded/locked	7	41.2
Unloaded/unlocked	6	35.3
Loaded/locked	2	11.8
Loaded/unlocked	5	29.4
**Firearm behaviors in past 30 days** ^d^ (*n* = 17)		
Handled a gun	8	47.1
Fired a gun	1	5.9
Carried a loaded gun	1	5.9

*Note*. Responses not endorsed by any participants generally are not included. For study eligibility, all participants had personally owned firearm(s) and/or lived in a household with firearm(s) present at some point since separating from military service.

^a^ If a participant indicated both current and past firearm access, they were categorized as current for this variable, which was mutually exclusive.

^b^ Included mental health providers (*n* = 19; 47.5% of overall sample), primary care providers (*n* = 4; 10.0%), and other providers (*n* = 1; 2.5%).

^c^ Response options not mutually exclusive.

^d^ Included “much” (*n* = 10; 62.5%), “somewhat” (*n* = 4; 25.0%), and “a little” (*n* = 0) safer.

## Results

### Thematic analysis

Three broad themes were identified: (1) women Veterans’ backgrounds, personal experiences, and beliefs shaped their perspectives on firearms and firearm LMC; (2) previous experiences with LMC were viewed as positive and collaborative or negative and with stigmatizing interactions that resulted in uncertainty and fear; and, (3) participants’ preferences for firearm LMC included what, when, why, how, and with whom to discuss firearms within the context of LMC with healthcare providers. Together, these themes provide a holistic, integrated appraisal of women Veterans’ perspectives, experiences, and preferences regarding firearm LMC.

### Theme 1: Women Veterans’ perspectives of firearms and firearm LMC

#### Shaped by military service and identity

Women Veterans described factors that shaped and influenced their perspectives on firearms and firearm LMC. For many women Veterans in our sample, military service was a formative event in their lives, and their experiences as Service members influenced their current beliefs regarding firearms. One woman explained how her years of military service had oriented her to “be this sort of protective figure with a gun, where you’re willing to give up your life and protect others with this lethal means,” and that “when you’ve been a Soldier, it’s such a part of you, having a gun.” This identity, however, was held in tension: many participants acknowledged that this familiarity with firearms in a protector role could yield destructive results. One participant stated that firearms can be “a threat to yourself and other people, too… it’s a really complex thing.”

#### Military sexual trauma, betrayal and mistrust

As noted previously, the majority of participants (85%) screened positive for MST, and many described how their MST experiences had shaped their current use of firearms (e.g., maintaining access to a firearm for protection) and perspectives on firearms and firearm LMC. For example, when asked about her reasons for maintaining access to a firearm, a Veteran who had experienced MST stated, “If I’m clinging to my firearm, that’s my protection, that’s my safety, that’s my safety blanket. I think I’m gonna fear discussing it because you might take it away from me.” In contrast, a few women who had experienced MST described not wanting any access to firearms due to the psychological impact of MST and the resultant suicide risk, although this was not common. Recalling her previous experiences of MST and IPV, one Veteran stated, “It’s been helpful for me not to be around guns…. not owning a gun has saved my life these last 10 years. Because there’s been times where I was so sad that, if I’d had a gun, if I would have been weak for that split second, it would take all my strength to not matter.”

Notably, among women who had experienced MST, associated feelings of betrayal and mistrust had a profound impact on their willingness to discuss firearms with their healthcare providers. As one Veteran described, survivors of MST are “automatically going to be more distrustful of the system because they’ve not gotten the proper care they deserve, they been lied to, manipulated, cheated on… one hundred percent they are going to be distrustful of the whole system.” This sense of institutional betrayal led many women in our sample to be distrustful of discussing firearms with VA providers. Drawing parallels between previous feelings of “constantly battling” and feeling as though “her career may be on the line” after seeking care while in the service, one Veteran explained how this manifested as continued apprehensions of disclosing her firearm access to her VA clinician. “I already had my own fears of what was really in my head with the job that I did [in the military]…But now I have the fear of my clinician breaking confidentiality and this becoming bigger than I was prepared for.” One participant who also described being reticent to discuss firearms with her healthcare providers stated that she was distrustful of the military, and by extension the VA, because “they’re not there to serve me, they’re there to serve the government.”

#### Spouses and partners

Women Veterans also described their relationships with their spouse or partner as another factor that influenced their firearm access, including how and where firearms were stored, as well as if firearms were stored locked. In some instances, this was a collaborative process, in which both parties had similar perspectives and beliefs about firearms in the home. For example, one Veteran described a conversation with her partner about her reasons for locking her partner’s firearm: “I explained to her that I am bipolar, that I am impulsive, and I explained my reasons [for locking up her firearm]. And she accepted them. She understands that, in order to be safe in this house, I need to feel safe in this house.” For some women, however, this process was imbalanced, whereby their partner or spouse had exclusive control about firearms in the home. “My ex did everything without consenting me and made all of his decisions independently of what I thought [about the firearms],” one Veteran stated. Another woman who had experienced IPV recalled her husband’s response to her objections regarding how he had been storing his firearms, given that they had a young child in their home: *“*He said they [the firearms] were safe, she [their daughter] couldn’t get up there [and access the firearms], so I should just shut up and leave it alone and quit poking at it.”

#### Living space: Children, rurality, upbringing

Closely related to the aforementioned findings, women Veterans’ living space, including *with whom* and *where* they lived, also shaped their perspectives on firearms and firearm LMC. In addition to whether they lived with a partner, many women conveyed how the presence of children (e.g., their own or their grandchildren) in their home changed how they thought about, stored, and used firearms, as well as their willingness to engage in firearm LMC. In describing the need for secure firearm storage, one woman noted that she had been “adamant about that because we had small children…I just didn’t want any accidents. That was my worst nightmare.” She described allowing her husband to have firearms in the home “as long as you can promise me our children will never shoot each other or shoot themselves by accident…my biggest fear was one of my children getting ahold of a gun.” Consequently, all firearms were locked securely in a safe.

For some women living in rural areas, firearm ownership was culturally normative: “You’re just like everyone else in the rural area–if you live in the woods, you have a firearm…it’s almost automatic where I’m from to have a firearm. And to not have a firearm, you feel almost naked.” Many women Veterans noted that growing up in a “gun culture” or being around others who valued firearms in similar ways also shaped their beliefs regarding firearms. Some women explained that they grew up in “gun totin’ families,” that “you don’t go into my family’s house and ask them if they have a gun–you ask them, how many they got?”

#### Personal history of suicidality

Finally, women Veterans described how their personal histories of suicidal thoughts or attempts had shaped their perspectives of firearms and firearm LMC. Due to such experiences, many were cognizant of the danger firearms might pose to themselves and expressed that they would be willing to engage in firearm LMC. For example, one woman had previously believed that restricting her firearm access was “ridiculous.” While hospitalized for a suicide attempt, however, a conversation with a healthcare provider had completely changed her thinking, despite her earlier belief: “It really wasn’t until a healthcare provider at the mental health hospital said, ‘You know what, you really don’t need to have a gun in your house’…I realized–I think that’s correct, and it really changed my thinking.” Some other women Veterans expressed having similar beliefs after a suicidal crisis or suicide attempt. One expressed that she did not trust herself to have a firearm because “it’s too easy. Too spontaneous.” Another explained that not having a firearm accessible is “just logical. If I’m sad enough that I wanna die, and that’s the quickest way to die, why would I keep it near me?”

### Theme 2: Women Veterans’ prior experiences with LMC

As stated previously, only half of women Veterans in this sample could recall ever having *any* conversation with a healthcare provider regarding firearms, despite all being in VHA care and having a history of suicidal ideation and/or attempt(s). Women who had experienced such conversations described positive or negative experiences discussing firearms with healthcare providers, detailing factors that had facilitated or hindered previous attempts to engage in firearm LMC. Many of the factors described here, while specifically framed around firearm LMC, are nonetheless largely applicable to general clinical interactions women Veterans have experienced when receiving VHA care. In relaying these experiences, women Veterans revealed that many aspects that constitute a positive or negative firearm LMC experience closely mirror what would make for a positive or negative clinical experience *generally*. Detailing these past experiences can reveal ways to better engage women Veterans in future firearm LMC discussions.

#### Negative experiences and barriers

Women Veterans who had participated in LMC and had negative experiences with firearm LMC described aspects that made such experiences negative. For example, many felt it was not comprehensive enough. Many were offput by the impersonal “checklist” approach to these conversations, where providers “ran through the motions,” rapidly working through a standard set of basic questions regarding firearms, such as simple “yes” or “no” questions regarding if they owned a firearm or if it was stored loaded and/or locked. Many women described experiences discussing firearms with their healthcare providers that were not perceived as productive or positive, describing apprehensions and fears about disclosing information about their firearms which then impeded LMC conversations. For example, numerous women described how they had been (and still would be) apprehensive and unwilling to engage with a provider in discussing firearms and their mental health out of fear that it would result in involuntary hospitalization. “You keep those things secret,” one Veteran explained, not wanting to disclose her suicidal ideation and firearm access to any healthcare provider, stating how “even if I had access, I’d tell ‘em I didn’t have access.” “You don’t tell people that or else they put you in a mental hospital. You don’t discuss it because the moment you do, you’re institutionalized.” Many also expressed concerns about having their firearms seized if a provider deemed them to be a threat to themselves or others. One woman explained that she didn’t disclose whether she owned a firearm during a mental health crisis because “the only thing running through your head in a crisis is ‘these people are going to call the police on me, they’re going to send someone to take my guns and take away my rights, they’re gonna put me in the hospital, they’re gonna hold me against my will.’” While many women described concerns regarding the implications of truthfully disclosing their firearm access, which resulted in them not disclosing their firearm status, none described experiencing any adverse actions, such as seizure of firearms, as a result of discussing their firearm access.

Hesitations regarding engaging in firearm LMC were borne from feeling that their providers were cold, distant, and impersonal, lacking a personal touch that conveyed that they could be trusted. Many women felt that they were not heard during healthcare visits and that they were treated as “just another number, another science project, a case study… that human factor wasn’t in the room…I’m not gonna sit here and be vulnerable and bleed all over the floor and you just sit there and stare at me.” Another woman expressed that, if she felt unheard, “I’m getting frustrated with them, I’m just going to take the easiest way out. Just tell me what you’ve got to say, and I want to get out of here.” Such feelings further disinclined women Veterans from engaging in firearm LMC. Perhaps more detrimental, many women left encounters that had entailed discussing firearms feeling as though the provider perceived them to be irresponsible or dangerous with their firearms: “They also suggested that [I was irresponsible], asking me why I have it [the firearm] in the first place…I didn’t like it. I didn’t like the fact that they were questioning me about why I have it.”

#### Positive experiences and facilitators

Positive LMC experiences were predicated on perceived trust, rapport, mutual understanding, a genuine connection, and the perception that the healthcare provider was discussing firearms with them because they cared about the Veteran, rather than simply as part of routinely “running through the motions.” Providers being attentive, thoughtful, and demonstrating compassion and genuine care for Veterans’ concerns also fostered positive experiences discussing firearms. For example, this could include conversations feeling more like “talking to a trusted friend rather than a clinician,” where a provider “doesn’t just jump right into the conversation…they start simple with ‘how are you feeling today?’” Veterans, particularly those who had experienced MST, also preferred providers who were cognizant of their needs, such as “maintaining some distance, he didn’t make any sudden moves, he respected my bodily autonomy.” Attention to these needs engendered trust and rapport, leading many Veterans to feel more at ease and willing to discuss firearms. Veterans described how providers being upfront and direct about their intentions, communicating clearly *why* they were asking firearm questions, and noting exceptions to confidentiality assuaged their fears and promoted trust. “Be upfront and just impartial, like ‘Hey, we have to ask you these questions. We’re disclosing that if you say X, Y, Z, we’re going to have to report it,’ kind of stuff,” one Veteran explained. “Just a nonbiased, nonjudgmental meeting, the facts… As long as [they] knows that they’re not gonna take it away just cuz they’re honest about it.” One woman explained, while “there are some individuals who think you are trying to take their gun away from them,” such conversations are necessary and need to be direct: “‘Do you have access to firearms?’ And just open up that conversation.”

### Theme 3: Women Veterans’ preferences for future LMC

Women Veterans described their preferences for how providers should engage Veterans in firearm LMC in the future. As described earlier, many of these preferences reflected their personal backgrounds, previous LMC experiences, and current firearm beliefs. Importantly, echoing earlier sentiments, while many of these preferences were focused on firearm LMC, many also related to other, more general aspects of clinical care. In presenting their firearm LMC preferences, women Veterans again made a link to their preferences for future engagement with VHA broadly. In particular, an overarching notion of feeling welcome and respected within VHA appeared paramount to engagingt in VHA services and firearm LMC specifically. That is, once engaged in VHA services with a trusted provider and rapport present, meaningful conversations surrounding firearm LMC could occur. Understanding these preferences–even those that are more pertinent to general VHA care–is vital, as meeting these preferences can further encourage women Veterans to seek VHA care and thus, subsequently engage in firearm LMC.

**Why are (or Aren’t) LMC conversations important?** Women Veterans provided their thoughts on why (or why not) firearm discussions are important to have. Many participants expressed that firearm access ought to be limited during mental health crises. One explained that “people that are suicidal, they are less likely to act on it if they don’t have the means, the method, or the motivation. So if someone is having a difficult time in their life and they don’t have access to a firearm, that takes away a lethal means.” Limiting access during times of elevated suicide risk was seen as valid and necessary for suicide prevention, albeit complex.

Some women Veterans expressed concern that if firearm access is restricted, many will find alternate means instead. A Veteran wondered “are they going to take the kitchen knives too? I mean, there are a lot of different ways. You could just buy a bottle of sleeping pills or something at the store and do it that way.” Some also questioned whether suicide could be prevented outright, arguing that a motivated individual could not be stopped. “None of those things–the lock box, the firearm lock–none of those things will prevent it,” one woman said. “They won’t prevent it. It will just slow it down by a matter of minutes or however long it takes them to remember where they hid the key. But again, if that’s how you’re feeling, there’s nothing that will prevent it.”

Furthermore, some Veterans expressed concerns about the political and constitutional implications of firearm LMC, worried that depriving someone of their rights and property required a measured approach. This was truly “a sticky question,” one participant stated, explaining how “I don’t want to ever say if you’re a female Veteran and you’ve identified yourself as a victim of MST or PTSD or anything else, then you can no longer own a gun. That’s not okay with me. I don’t want it to ever be an additional stigma or negative because you sought help and now you can’t protect yourself.” Reiterating this point, another Veteran believed that while “sometimes people reach a certain mental point, they need help to protect them from themselves, but at the same time, I also don’t believe in confiscating people’s firearms either.”

**How should LMC conversations be discussed and framed?** Participants then described how conversations about firearms should begin and how provider framing and tone matter. Women Veterans felt that it was best if providers made firearm conversations routine and normal, calmly and dispassionately asking about firearm access. Participants noted that making these discussions just another part of a broader assessment could normalize them, whether through part of a basic questionnaire or a quick conversation with their providers. Another piece of this normalization comes from framing the conversation around safety in general, not specific to firearms, but encompassing other means and expanding the scope to include the safety of others around the Veteran. A participant stated that providers should “preface it with the concerns of safety. You know, ‘For safety concerns, we like to ask if you have access to any firearms. And it’s not only the safety for you as a Veteran, but the safety of those around you.’” “I think if there was a better way of communicating, to say ‘It’s alright to not be alright,” one Veteran began explaining, “‘We’re gonna get you the resources you need, but in the meantime, we’re going to create a safety plan and that safety plan is gonna be a temporary removal of your access to firearms and your access to other weapons, bows and arrows, knives, whatever.’”

Furthermore, participants also noted a need for providers to address IPV when attempting to discern firearms access among women Veterans. They explained a need for providers to be attuned to body language, posture, and other non-verbal clues that may indicate their patient may currently be experiencing IPV. Veterans described how providers ought to “watch their body language and see how they react. If they look down, if they’re visibly tense, if they just shrink back, if they look away from you, if they break eye contact–there may be some issues around guns in the house and they’re afraid to say something.” Discerning these non-verbal cues can be especially vital in instances in which a Veteran is accompanied to clinical visits with a partner, or may not feel empowered to broach the subject themselves. Having these skills would thus enable providers to effectively engage women Veterans in discussing firearms, as well as to understand ways in which firearms may be used to further enact IPV.

**What should be discussed in LMC conversations?** Women Veterans expressed that firearm access should be assessed by not only discerning if the Veteran has access to a firearm, but also their storage practices (e.g., loaded, unlocked) and the function of ownership (e.g., protection, hunting). Women Veterans recommended a series of additional, deeper probes that they felt more comprehensively and holistically appraised firearm access as important to help limit firearm access, when appropriate, and prevent firearm suicide. One woman Veteran suggested that, in addition to *generally* asking about firearm access, providers ought to ask specifically about the Veteran’s sense of safety being in a home with a firearm. “I think something that’s important to ask is not just ‘Do you have guns?’”, one Veteran explained, advocating for providers to go deeper, recommending that they also ask: “‘Does anyone else in the household have guns? Does your significant other have a gun? Has he or she ever threatened you with a gun or tried to intimidate you?’ Because no one ever asks that, they just ask ‘Do you have access to firearms?’”

Women Veterans also expressed that it is important for providers to assuage women Veterans’ fears that disclosing firearm ownership would trigger seizure of their firearms, involuntary institutionalization, or permanently marked VHA medical records. Describing this latter concern, one Veteran was cautious regarding whom she discussed firearms with, indicating that “the only notes that go in my file are those of the [non-VHA] psychiatrist; the VA providers cannot see my notes from the Vet Center. So that provides me with another layer of trust that they’re not [accessible], I can be the conduit of my records, I have control of that piece of it.” Further, many women Veterans described that, in order to discuss firearm access with their healthcare providers, they wanted “guarantees that it would be confidential and that there would be no impact on my future ability to buy firearms, my future ability to use firearms, my career” and “assurances that it wouldn’t affect their future ability to hold a security clearance or work in police work…I think that would be crucial…” Such concerns are consistent with other, similarly charged and potentially sensitive clinical issues, and underscore the need for precise framing and language on the part of clinicians to educate and assuage these fears.

**Who should initiate LMC conversations?** In terms of *who* should initiate LMC conversations in clinical settings, women Veterans placed great emphasis on this being *any* healthcare provider with whom they had a trusting, longstanding relationship. “I would only have concerns if I didn’t trust the person,” one Veteran explained when asked what would make her more willing to talk to a healthcare provider about her firearms. “You can tell if somebody really cares about you or not, or if they’re just asking questions because they have to be there.” Further–and perhaps related–women Veterans also felt that healthcare providers who understood military culture, terminology, hierarchy, and had military experience were better equipped to understand the needs of Veterans and more able to discuss firearms. Explaining this need for shared language and understanding, one participant conveyed the following about working alongside other women Veterans in group therapy, “[women Veterans] weren’t picky about whether or not it was a man or a woman. They were picky about the fact that the people they were talking to didn’t have a background in the society, the military society, the chain of command, what you learn in the military and the state of mind.” She continued to emphasize how not having this competency can hinder engaging with Veterans because if “they don’t know the terminology and then they get thrown in a group and somebody is using terminology they don’t understand, and they ask what it is, and that will shut the group down in a heartbeat.”

However, some women Veterans expressed a preference to engage in LMC with women providers. For example, when asked who she would feel more comfortable discussing firearms with, a Veteran stated: “I would probably prefer a psychologist–specifically a female versus a male.” Similarly, another woman expressed how, given the impact of prior trauma on her decisions to own and increase access to firearms, “Having a female therapist, especially when it comes to any kind of sexual assault or abuse, it’s just easier to talk to a woman than a man because typically, it’s the man who is doing the abusing.”

**Where should LMC conversations occur?** While some Veterans suggested that trusting, caring, and respectful relationships can occur anywhere, many women Veterans were quick to express some hesitations about discussing firearms with providers in VA settings. Some, particularly those who had experienced MST, expressed that the physical space within VA clinics made them less likely to be engaged in VA healthcare: “I experienced military sexual trauma, so it was hard being in the VA, being in a setting that [was] primarily male and knowing that one in three women, and actually, it’s more like one in two women have experienced sexual harassment or even sexual assault. And sitting next to a man and wondering, well, are you someone that raped a woman or beat her up or made disparaging comments? I still wonder if the guy I’m sitting next to or standing next to in line has done that.” One participant shared her anxieties about VA facilities, noting the lack of “privacy and atmosphere, and if you’re walking into a sterile environment, it just feels very clinical, people are psychoanalyzing you. Are they really interested in helping you, or are you just a lab experiment?”

Additionally, many participants described being dismayed regarding their perception of the lack of women-specific groups, spaces, or specialty care available, and accordingly, appeared to be disinclined from engaging in LMC in these settings. One Veteran conveyed her frustration and discomfort with mixed-gender support groups: “For a long time, I didn’t want to go to groups with other male Veterans because my story and my stress was so different.” Women described being more willing to engage in firearm discussions in groups comprised solely of other women Veterans because “there’s a shared experience, and it’s sort of like a sisterhood…if one of your sisters is in danger, you want to be there for her and prevent her from doing something that you can’t undo.” Similarly, another stated that “instead of giving me the locks or offering the firearm locks, get me into a group with other women Veterans.” A third woman echoed this, noting how such groups “help as far as talking about firearms because I don’t know what other women do…I think having support, like a peer support is just as important as having the mental health therapist.” Thus, the ability to engage in firearm LMC with fellow women Veterans, in a setting that felt safe and was specific to women Veterans, was a preference of some women Veterans for engaging in LMC in VA healthcare settings.

**When should LMC conversations occur?** Regarding when LMC discussions should occur, women Veterans again stressed the need for them to feel natural and routine, not something for a provider to “make a big deal out of.” Veterans were generally agreeable to receiving questions about firearm access when posed alongside assessing for suicide risk or in the context of general mental healthcare. Importantly, this does come into tension with a concern raised earlier by women Veterans: while it is necessary for LMC discussions to feel routine and normal, such conversations need to feel compassionate and borne from genuinely caring about the individual and her well-being. If suicide prevention conversations about firearms become too common, they can lose their impact and again feel like a “checklist” approach that many felt lacked precision and care. One woman Veteran described how this occurs, stating that “I know you guys are trying to help, but it’s become a joke among Veterans that if you go to the VA, they’re going to ask you if you’re suicidal regardless of what you’re there for.”

A summary of findings (i.e., subthemes and key points), derived from participants’ statements, as well as our suggestions regarding clinical implications of these findings, can be found in Table 4.

**Table 4:** Key points and clinical implications for firearm LMC with women veterans.

Subtheme	Key Point(s)	Clinical Implication(s)
**1. Firearm Perspectives **		
Shaped by military service and identity	Military service and identity shape firearms thoughts and beliefs.	Understanding a Veteran’s military service may bolster rapport and provide insights into the context of their firearm beliefs and behaviors.
Military sexual trauma, betrayal and mistrust	Military sexual trauma experiences may lead to firearm ownership, unsafe storage practices, and a desire to maintain firearm access. Conversely, military sexual trauma may lead to not wanting access to firearms due to the psychological impact of the event(s).	Assess for MST (and betrayal) experiences and understand that such experiences may deter some Veterans from disclosing their firearm access. Acknowledge that trust and rapport may bolster firearm LMC discussions.
	Perceived betrayal stemming from military sexual trauma experiences may deter Veterans from disclosing firearm ownership and lead to mistrust of VHA.	
Spouses and partners	Spouses and partners can influence if and how firearms are owned, stored, and used.	Spouses and partners are underutilized resources in firearm LMC; engaging them (if possible and when appropriate to do so) may be key to strengthening LMC for women Veterans.
	As women Veterans are *less likely* (than men Veterans) to own firearms themselves, the influence of spouses and partners may be more pronounced.	
Living space: children, rurality, upbringing	Presence of children/grandchildren in the home can be pivotal to storing firearms more securely.	Understanding Veterans’ living space, including the presence of children, cultural background, and geographic location, can provide important contextual information regarding potential facilitators and barriers to firearm LMC.
	Cultural factors, like rurality and upbringing, also influence women Veterans’ firearm perspectives.	
Personal history of suicidality	Veterans with a history of suicidality may make be *less willing* to own a firearm and may have more desire to reduce their firearm access.	Assessing a Veteran’s history of suicidal thoughts and behaviors, including the role of firearms in such experiences (e.g., planning, attempts), is important and may provide important information regarding a potential motivator for reducing firearm access.
**2. LMC Experiences **		
Negative experiences and barriers	Impersonal, dismissive, and cold interactions; feeling unheard, minimized, or treated as “just another patient.”	Affirm commitment to providing high-quality personalized care; be responsive to individual needs and preferences.
	Fear of firearm seizure, involuntary hospitalization, or other legal consequences in disclosing firearm access.	Acknowledge and address fears by clarifying *how*, *when*, and *why* discussing these matters; be upfront and honest about the limited conditions in which those circumstances *could* occur. Respond to concerns regarding firearm seizure or involuntary hospitalization.
Positive experiences and facilitators	Trust, rapport, and mutual understanding; genuine connection and care; active listening; being attentive to one’s concerns and needs.	Provision of high-quality personalized care, including taking time to understand and be attentive to unique needs and preferences, may bolster firearm LMC.
	Providers clearly communicating the rationale for asking about firearms.	Communicate why firearms are being discussed; emphasize that the Veteran’s safety is the key concern.
**3. LMC Preferences **		
Why is firearm LMC important?	Many acknowledged the import of firearm discussions because limiting access to firearms during times of suicidal crisis can prevent suicide.	Reaffirm the importance of discussing firearms by linking it to suicide prevention.
	Others believed that even if you limit access to firearms, many will find alternative means of suicide; they also expressed concerns about the political and constitutional basis of LMC.	Provide education regarding the link between firearm access and firearm suicide. Dispel misconceptions about means substitution.
How should firearm LMC conversations be framed?	Conversations should feel normal, dispassionate, and standard.	Calmly and honestly inquire about firearms. Preface firearm questions with normalizing the reason(s) for asking such questions.
	Frame around safety of the Veteran and those around them.	Broach the discussion around the issue of safety.
	Assuage fears of firearm seizure, involuntary hospitalization, and legal consequences.	Be honest about the intent of the discussion and the specific circumstances that could trigger rare instances of firearm seizure, involuntary hospitalization, or legal consequences.
What should be discussed during firearm LMC conversations?	Only half of women Veteran participants reported *ever* having had discussions regarding firearms with a clinician.	There is likely a substantial portion of women Veterans who have never had firearm discussions with their healthcare providers. Initiating trauma-informed, personalized discussions regarding firearms is warranted.
	Simple checklists with “yes” or “no” questions were deemed insufficient; women Veterans wanted questions that probed for contextual factors, such as intimate partner violence.	Broaden the scope of firearm LMC probes to assess for intimate partner violence; does the Veteran feel safe in the home, have they been threatened with firearms; do they have a say in how firearms are used and stored?
Who should initiate these conversations?	Trust is paramount and perhaps more important than defining who is best- equipped to start these conversations.	Provider further education to clinicians on military structure, terminology, and language, to facilitate building trust, connection, and rapport with Veterans.
	Preference for women providers, peers (i.e., other women Veterans), and providers with military cultural competence.	If appropriate and feasible, provide the option for women Veterans to speak with women providers, if one’s own identity/background is hindering firearm LMC conversations.
Where should firearm LMC conversations occur?	Concerns about VHA facilities, including perceived lack of women-specific spaces and concerns about sexual harassment.	Create and promote more women-centered support groups, networks, and programming. Continued VHA efforts to prevent harassment at VHA facilities are important to ensuring that women Veterans feel comfortable obtaining services and discussing LMC in VHA.
	Strong support for more women-specific support groups, networks, and programming.	Providing LMC in groups comprised of women Veterans is a potential opportunity worth exploring in future research.
When should firearm LMC conversations occur?	Conversations should occur regularly and feel like a normal line of inquiry.	Firearm LMC conversations should be normalized and integrated into other standardized questionnaires.
	Yet at the same time, firearm LMC conversations should not feel *too* normalized, at the risk of feeling like a “checklist” of items not worth caring about.	These questions and subsequent conversations still need to feel personalized to the individual.

*Note*. LMC = lethal means counseling.

## Discussion

Firearm-related suicide among women Veterans is a profound public health concern. Optimizing firearm LMC for women Veterans requires understanding and consideration of gender-specific needs, experiences, and preferences. With that in mind, we conducted the largest qualitative study of women Veterans to date to explore how understanding such factors can facilitate gender-sensitive firearm LMC with this population. Our findings underscore the unique backgrounds and lived experiences of women Veterans that influence their firearm and firearm LMC perspectives and preferences. Women Veterans described their experiences discussing firearms with healthcare providers, detailed preferences for how firearm discussions ought–and ought not–be broached, and revealed ways in which firearm LMC can be optimized for at-risk women Veterans. Curating effective and satisfactory LMC strategies requires attention to women Veterans’ individual histories, previous experiences with firearm LMC, perceptions of VHA facilities (e.g., perceived sense of safety), and stated preferences.

Our findings suggest that, to optimize firearm LMC discussions with women Veterans, providers should work with their women Veteran patients to explore their histories, with a focus on experiences that may impact Veterans’ perspectives of firearms and willingness to engage in firearm conversations. Injuries and traumas sustained during service, including those related to MST as well as IPV, comprised some of the experiences that impacted women Veterans’ perspectives on firearms and firearm LMC. For example, woman Veterans who had experienced MST tended to have firearms for personal protection, tended to prefer women providers for firearm LMC, and described betrayal and mistrust as MST-related concerns that had deterred them from discussing firearms with healthcare providers. Acknowledging these prior experiences (e.g., MST, betrayal, mistrust) and their potential impact on women Veterans’ willingness and comfort with discussing firearm access and implementing different means of firearm storage when their suicide risk is elevated may enable providers to better understand women Veterans’ reasons for having firearms or storing them unsafely, and accordingly, may facilitate tailoring firearm LMC within a trauma-informed framework. While women Veterans are routinely screened for both MST and IPV within VHA, such experiences are vastly underreported [44–47]. Thus, assessing for a history of MST, IPV, and other types of interpersonal violence across the lifespan in the context of firearm LMC discussions may be particularly important to framing firearm conversations.

Furthermore, recognizing the influence of women Veterans’ cultural backgrounds and upbringings is relevant to firearm LMC. By assessing not just *if* a Veteran owns a firearm, but the firearm’s use, meaning, purpose, and function, providers can gain insights into what storage recommendations or changes may be best for the Veteran, as well as potential barriers and facilitators to doing so. Willingness to adopt certain storage methods may be different for a woman Veteran whose firearm ownership serves as an important reminder of her military service, compared to a woman Veteran who maintains access to unlocked, loaded firearms as a means of protection in the context of prior IPV or MST. Providers can work with women Veterans to consider different in-home or out-of-home storage options that work best for their own personal circumstances (e.g., enhancing safety through means other than a firearm) and preferences. To improve Veterans’ willingness to disclose their firearm access, providers can also re-frame these discussions by carefully explaining their rationale for asking such questions and describing how such information will be used at the beginning of such conversations.

We identified an important tension in themes that is relevant for implementing firearm LMC interventions with women Veterans. While some women Veterans indicated that making LMC discussions routine, even through standard questionnaires, might serve to normalize conversations and make them more acceptable, others preferred that such discussions be delivered in a personalized fashion, rather than through a “checklist” approach, or, as one perceived, as “running through the motions.” Women Veterans also desired that LMC conversations be initiated by a clinician who is trusted and within the context of a long-standing relationship. This is consistent with prior research in which Veterans have underscored the import of trust to firearm conversations [17], as well as suicide risk assessment and disclosure [48, 49]. Trust has also been underscored as important in the context of disclosure of military sexual trauma [50] and IPV [51, 52], as well as treatment of associated sequelae. Variability in individual preferences, however, has been identified in prior studies regarding firearm LMC [53, 54], and may pose challenges to implementing firearm LMC interventions and broader suicide risk identification initiatives that rely on highly standardized or protocolized assessments. This is particularly relevant for the care of women Veterans, where screening for firearm access and prompts to initiate LMC within VHA often occur within the context of an enterprise-wide suicide risk screening and evaluation protocol. Additional work is needed to better understand the acceptability, efficacy, and effectiveness of firearm LMC when delivered according to protocols, in comparison to clinician-initiated discussions.

Only half of women Veterans in our sample indicated that they had engaged in a firearm LMC discussion with a healthcare provider, suggesting a need for increased firearm LMC. Moreover, our findings suggest that the purview of firearm LMC questions with women Veterans needs to be enhanced. Instead of solely asking standard firearm LMC questions, providers could additionally ask more broadly *who* owns the firearm, if the Veteran feels safe in the home with this person and the firearm, if they have any say or agency in how the firearm is stored and used, and if they have been threatened or intimidated with it. Such questions are especially pertinent to women Veterans given knowledge of the high proportion of women Veterans who live in households with a firearm that is not their own (typically a spouse or partner’s) [17, 55]. Doing so can provide a more holistic appraisal of household firearms and the relation of those firearms to the Veteran. In fact, recent survey data suggest that only 16–20% of women consider themselves the owner of firearms accessible in the home [56]. Further, IPV is highly prevalent among women Veterans [57], which may render household firearms particularly risky to women Veterans.

Additional training and education for healthcare providers can facilitate this broader line of inquiry, as well as continued (or increased) use of firearm LMC with women Veterans. Such training is needed given what is known about healthcare providers’ lack of knowledge and familiarity with firearms and storage devices [12, 58, 59], military and firearm cultural competence [60–62], and data suggesting that most firearm owners–including Veterans–are open and willing to discuss firearms in certain contexts [63, 64]. It is also important for healthcare providers working in settings where women Veterans comprise the majority of patients, such as reproductive health settings [49]. Concurrent efforts to increase the general application of firearm LMC techniques, while also equipping providers with knowledge regarding barriers and facilitators of using firearm LMC with women Veterans, can normalize firearm questions that occur as part of suicide risk assessments and enable providers to provide gender-sensitive firearm LMC to women Veterans in accordance with Veterans’ needs and preferences. Given that participants voiced concerns that disclosing firearm access during clinical encounters may impact their ability to legally possess a firearm or that such disclosure may result in firearms being seized (a sentiment demonstrated in other qualitative studies) [11, 65], it is essential to ensure that clinicians are well-versed in the rare instances in which seeking mental health care may have the potential to impact patients’ firearm rights. Indeed, while participants in this study (and others) maintained apprehensions about disclosing firearm ownership status out of fear that doing so would result in involuntary firearm seizures or restrictions [11, 65], such instances are rare; to date, only two states enable healthcare providers to act directly as petitioners for extreme risk protection orders (Maryland as of 2018; Colorado as of 2023) [66, 67], and many healthcare systems (including the Veterans Health Administration) do not report directly to the National Instant Criminal Background Check System (NICS) [68]. Importantly, the disclosure of firearm access or ownership in itself is not a factor that impacts reporting on federal background check forms. Broaching women Veterans’ concerns regarding discussing firearms and informing them of the relative rarity and limited scope of involuntary firearm seizures and restrictions is important.

Lastly, when describing their preferences for firearm LMC, women Veterans discussed some concerns about firearm LMC occurring in VHA facilities, due to perceived lack of women-specific spaces and concerns about sexual harassment. They also described a desire for more women-specific support groups and programming. These concerns and preferences have been noted in previous studies of women Veterans [51, 69–71]; for example, the desire for connection appears particularly important to women Veterans, who have noted connection as necessary to feel as though they can let down their guard and be vulnerable [71]. Thus, our findings suggest that women Veterans’ concerns regarding sexual harassment at VA facilities, as well as their preferences for women-specific therapy groups, extend to a variety of domains relevant to suicide prevention, including as a potential barrier (sexual harassment) or facilitator (women-specific groups, trust, sense of safety) to firearm LMC. Understanding how to incorporate peer support into firearm LMC for women Veterans may be an important next step, given women Veterans’ preferences regarding firearm LMC. Moreover, our findings suggest the continued import of trauma-informed principles, such as creating a safe space, for women Veterans as part of firearm-related suicide prevention [14].

There are important limitations to note. First, although qualitative studies generally are not intended to be generalizable [72], given our eligibility criteria, findings may not be applicable to women Veterans who never used VHA services or who never experienced suicidal ideation or suicide attempts. While LMC is most pertinent to women Veterans who have experienced suicidal ideation and attempt, women Veterans without a history of suicidal ideation or attempt may have differing perspectives on discussing firearms; understanding their perspectives may be important for elucidating upstream approaches to firearm-related suicide prevention. Future research is warranted to understand if such differences exist. Additionally, when recruiting for this study, we were transparent about our focus on firearms and suicide prevention; it is possible that in doing so, those with strong concerns about discussing firearms elected not to participate. Our sample was also predominantly comprised of those with lifetime trauma, MST, lifetime IPV, and positive PTSD screens. While this is a crucial group to focus on for suicide prevention, this may limit findings to women Veterans without such histories. Finally, our sample included low numbers and proportions of participants who were Hispanic, Asian American, and Pacific Islander, as well as those with children in the home; consequently, findings may be limited for these groups, and further examination is warranted.

Preventing firearm suicide remains paramount, and continued firearm LMC represents an important effort to prevent suicide. Optimizing this practice requires a gender-sensitive approach that includes broader, comprehensive assessment of women Veterans’ reasons for owning, using, and storing firearms. Understanding the unique backgrounds, personal preferences, and past experiences of women Veterans, including with respect to trauma and interpersonal violence, can enable healthcare providers to work collaboratively with their women Veteran patients to facilitate firearm LMS during times of elevated suicide risk; thereby reducing the potential for firearm injuries and deaths in this population.

## Supporting information

S1 Appendix Qualitative interview guide. Study qualitative interview guide, data collection instrument. (DOCX)
