# Supplementary material for: Women Veterans’ perspectives, experiences, and preferences for firearm lethal means counseling discussions
Source: PLoS One. 2023 Dec 6;18(12):e0295042. doi: 10.1371/journal.pone.0295042 (PMC10699600; doi:10.1371/journal.pone.0295042)
Supplement: S1 Appendix — (DOCX) [file pone.0295042.s001.docx]

# Aim 1 Qualitative Interview Guide

*As is conventional for qualitative research using semi-structured interviews, the exact wording of questions and probes will vary. The questions and probes listed here are representative of the general content to be explored. Not every question may be asked of every participant depending on interview flow and timing.*

*For this interview, I’ll be asking you some questions about your experiences and opinions regarding firearms and suicide prevention. There are no right or wrong answers to any of these questions. Rather, my goal is to understand your perspective and experiences. Before we begin, I would like to confirm –* ***do I have your permission to audio record this interview?***

**Context**

1. Please tell me a little bit about yourself, such as your military service, use of VHA care, and family.

Additional prompts: What types of healthcare have you received in the VA (e.g., primary care, mental health)? Who else lives in your home?

# Suicide Risk and Prevention among Women Veterans

1. When you think about women Veterans who die by suicide, what comes to mind?

Additional prompt: Why do you think some women Veterans die by suicide?

1. Do you think there are any differences in factors that lead to suicide between…
   1. Veteran women vs Veteran men?
   2. Women Veterans and women non-Veterans?
2. To your knowledge, when women Veterans die by suicide, what is the most common suicide method used?
3. How do you think women Veteran suicide can be prevented?
   1. What things do you would be helpful?
   2. What things do you think would not be helpful? What about harmful?

# Firearms

6. Can you tell me about the firearms that you currently or previously have had in your household [since adulthood]?

Additional prompts (*Modify wording to assess past firearms, as necessary)*

1. What kinds do you or other household members have? (e.g., handguns, long guns)
2. Who owns them?
3. Where are they located?
4. Who has access to them?
5. Why/when were they acquired?
6. How are they used?
7. Were there any additional firearms you have had access to or could access outside of your home that we have not already discussed? Please describe.

7. How are these firearms [described above in Q6] stored (or how were they previously stored)? Are they locked and loaded? What prompted these storage practices?

8. What was the process of deciding whether to have these firearms, how they would be stored, and who would have access to them?

Additional prompts: Who makes decisions about household firearms and how they are stored? How are these decisions made?

1. How do *you* feel about having these firearms and how they are stored?

Additional prompts: What aspects do you: …like? …dislike?

1. How do (or how have) *other* *household* *members* feel (felt) about having these firearms and how they are stored?

Additional prompts: What aspects do they :…like? …dislike?

Have you all ever disagreed about having firearms in your home or about

how they were stored? How was this resolved?

9. Have you ever considered storing them differently? Why or why not?

10. Have you or another household member ever changed how household firearms were stored? Please describe.

Additional prompts: What prompted this? What was that like: …for you? …for other household members?

**Firearm access when suicidal**

11. Please describe the most recent (or most serious) time in which you were suicidal (i.e., when you were having thoughts that you would be better off dead or were thinking about killing yourself). During that time or other times when you were suicidal… (*Ask for elaboration, as needed, and be sure to anchor these in when the participant was suicidal)*

1. did you have access to any firearms? If so, whose? How were household firearms stored?
2. did your beliefs and feelings about firearms change?
3. did you think about using a firearm as a means of suicide?
4. did your firearm use and access change?
5. did you or someone else change how household firearms were stored?
6. what else would have been helpful for getting through that time?
7. did you speak with any healthcare providers about household firearms?
   - 1. (If yes) Who did you speak with? What type of provider [e.g., mental health, primary care, ER, etc; VHA vs non]?
        1. What prompted this discussion?
        2. How did you feel about how it went?
        3. What was helpful about the discussion?
        4. What did you wish had gone differently or think could have gone better?
     2. (If no) Why not?
        1. If a provider had brought this up, how would you have responded?
        2. What would have been helpful? …not helpful?
8. did you speak with any household members or family members about household firearms?
   1. (If yes)
      1. Whom did you speak with?
      2. What prompted this discussion?
      3. What was helpful about the discussion? What did you wish had gone differently or think could have gone better?
   2. (If no)
      1. Why not?
      2. If a family member had brought this up, how would you have responded?
      3. What would have been helpful? …not helpful?
9. During this time, did you or other individuals make any changes to your firearm access?
   1. (if yes) Please explain.
      1. What was your goal in this (if any)?
      2. What helped to facilitate this change in firearm access?
      3. What barriers did you encounter?
      4. What would facilitate or further improve this process, if anything?

# Firearm Lethal Means Safety

1. How would you feel if a VA healthcare provider asked you about your access to firearms? Would you be willing to talk with a VA healthcare provider about your access to firearms?
   1. Please explain, including the circumstances you would you be willing to discuss your firearm access with a VA healthcare provider. What type(s) of providers or settings would be most helpful to facilitate this discussion? …least helpful? *[Explore provider type and settings]*
   2. How would you prefer for a healthcare provider to approach this conversation?

Additional prompts: What would you want the provider to say or do?

- 1. Would you prefer for anyone else to be there for the discussion?
  2. Do you have any concerns about discussing firearms with VA healthcare providers? If so, what would help you feel more comfortable discussing firearms with a VA healthcare provider?

1. Do you think women Veterans should have access to firearms when they are suicidal? Why or why not? What about when they are experiencing mental health concerns?
2. (If yes) What could be done to prevent suicide via firearms among women Veterans?
3. (If no) How should this be addressed? Additional prompt: What would be your preference for addressing this?
4. One suicide prevention recommendation is **limiting access to firearms** when people are at increased risk for suicide. Were you aware of this recommendation? What do you think of this?
   1. What would increase your willingness to do this if you were suicidal?
   2. What would stop you from doing this, or make it challenging to do, if you were suicidal?
   3. What would your partner, family, or other household members (if applicable) think about this recommendation?
   4. How do you think your healthcare provider(s) would respond to this recommendation, if you were suicidal?
5. If you were suicidal and were willing to temporarily reduce your access to firearms, who would you want to be involved? Who would you be willing to involve?

Additional prompts: How would you feel if family or household members were involved? How would you feel if a healthcare provider were involved? Which provider(s) would you be willing to talk to you? Would the type of provider (e.g., primary care versus mental health) make a difference? How so? What would support this process? What do you think could potentially get in the way of this process?

**Interpersonal Violence**

1. Do you think having a history of sexual assault or intimate partner violence influences how women Veterans feel about: …reducing their firearm access? … involving family members in firearm-related conversations or efforts? …talking to healthcare providers about firearm access? If so, what would be most helpful for addressing these concerns?

**Past Year / Recent Events**

1. Have any of your perspectives or experiences about firearms or suicide prevention changed in the past year or so – for example, in relation to recent events during this time? If so, please describe.

# Closing comments

1. Is there anything else you would like to share with our team, including about how to prevent firearm-related suicide among women Veterans?
